# Application of hydrogels in 3D tumor microenvironment modeling and drug screening for cervical cancer: A review

**DOI:** 10.1002/btm2.70147

**Published:** 2026-04-21

**Authors:** Ying Liu

**Affiliations:** ^1^ Department of Central Laboratory The Third Affiliated Hospital of Jinzhou Medical University Jinzhou China

**Keywords:** 3D tumor microenvironment, cervical cancer, drug screening, hydrogels, in vitro models, tumor biology

## Abstract

Cervical cancer, driven mainly by human papillomavirus (HPV) infection, remains one of the most common malignant tumors among women worldwide, posing significant challenges in treatment and drug development. Traditional two‐dimensional (2D) cell culture models fail to accurately replicate the in vivo tumor microenvironment (TME), especially HPV‐driven oncogenic signaling, immune contexture, and stromal interactions unique to cervical cancer, limiting their predictive value for therapeutic efficacy (Y. Liu, H. Ai. Comprehensive insights into human papillomavirus and cervical cancer: pathophysiology, screening, and vaccination strategies. *Biochim Biophys Acta Rev Cancer*. 2024;1879(6):189192). Hydrogels have emerged as promising biomaterials for constructing three‐dimensional (3D) tumor models due to their tunable physicochemical properties, excellent biocompatibility, and ability to mimic the extracellular matrix. This review focuses on hydrogel applications in 3D cervical cancer TME modeling, with an emphasis on recapitulating HPV‐driven biology, immune‐stromal crosstalk, and stromal interactions, emphasizing their role in simulating key aspects of tumor biology such as cell–cell and cell–matrix interactions, hypoxia, and drug resistance. Recent advances in hydrogel‐based 3D models for high‐throughput drug screening are critically analyzed, highlighting their potential to improve the precision of cervical cancer treatment and accelerate novel drug discovery. However, critical challenges including high cost, limited industrial scalability, technical complexity, and strict regulatory constraints remain to be addressed to realize their full translational potential. By integrating current research findings, this review aims to provide a theoretical framework and technical guidance for future studies focused on enhancing the physiological relevance of in vitro cervical cancer models and optimizing therapeutic strategies.

**Translational Impact Statement**

This review systematically consolidates cutting‐edge advances in hydrogel‐based 3D modeling of the cervical cancer tumor microenvironment, addressing the critical translational gap between conventional preclinical models and clinical therapeutic outcomes for HPV‐driven cervical cancer. It delineates the unique capacity of hydrogel platforms to recapitulate the disease‐specific immune‐stromal crosstalk, mechanical heterogeneity, and drug resistance phenotypes that underpin treatment failure, establishing a robust, physiologically relevant framework for high‐throughput preclinical drug screening across chemotherapy, targeted therapy, and immunotherapy modalities. By defining current technical bottlenecks and actionable translational development pathways, this work provides essential theoretical and technical guidance to accelerate the development of personalized cervical cancer treatment regimens and improve patient survival.

## 1 INTRODUCTION

Cervical cancer remains a significant public health challenge, ranking as the fourth most common cancer among women globally. Distinct from other solid tumors, cervical carcinogenesis is predominantly driven by persistent high‐risk HPV infection, which reshapes the local immune microenvironment and drives malignant progression. Its pathogenesis is intricately linked to the tumor microenvironment (TME), which encompasses a complex network of cellular and non‐cellular components that influence tumor behavior, progression, and response to treatment. The TME not only provides structural support but also modulates various biological processes, including HPV‐associated immune evasion, angiogenesis, and metastasis, making it a critical focus for developing effective therapeutic strategies.^1^ The interaction between HPV‐transformed cancer cells and their microenvironment is multifaceted, involving various cell types such as suppressive immune cells, cancer‐associated fibroblasts (CAFs) as key stromal components, which together create a unique niche that can either promote or inhibit tumor growth. The authors systematically investigated the crosstalk between tumor‐derived extracellular vesicles (EVs) and tumor‐associated macrophages (TAMs) in the cervical cancer TME using clinical specimens from 126 HPV‐positive cervical cancer patients and in vitro co‐culture models. This study revealed that HPV E6/E7 oncoprotein‐positive EVs secreted by cervical cancer cells could induce M2‐type polarization of macrophages, which in turn secreted TGF‐β and IL‐6 to promote the invasion and stemness of cancer cells, forming a positive feedback loop for immune suppression and tumor progression. This case directly demonstrates that the HPV‐driven immune‐stromal crosstalk in cervical cancer is a highly dynamic, multi‐cellular interactive process, which cannot be accurately simulated by traditional 2D monoculture models that lack the complex cellular components and paracrine signaling networks of the native TME.^2^

Traditional two‐dimensional (2D) cell culture models have been widely employed in cancer research; however, they fail to accurately replicate the heterogeneity and complexity of the TME found in vivo, and cannot capture the HPV‐driven characteristics, immune contexture, and stromal crosstalk specific to cervical cancer. These models can lead to misleading results, particularly in drug screening and therapeutic efficacy assessments, due to their inability to mimic the spatial organization and biochemical signals present in actual tumors. As a result, there is an urgent need for more sophisticated models that can better reflect the in vivo conditions of tumors.^3^ Recent advancements in three‐dimensional (3D) culture techniques, particularly those utilizing hydrogels, have emerged as promising solutions to this challenge.^4^ Hydrogels provide a supportive scaffold that can mimic the physical and biochemical properties of the ECM, facilitating more physiologically relevant interactions between tumor cells and their environment.^5^

Hydrogels possess several advantageous properties, including tunable mechanical strength, biocompatibility, and the ability to encapsulate various cell types and biomolecules. These characteristics make hydrogels ideal candidates for constructing 3D tumor models that closely resemble the native TME. By providing a more accurate representation of tumor architecture, hydrogels enable researchers to study cancer cell behavior, including proliferation, migration, and response to therapies, in a more relevant context.^6^ Moreover, 3D hydrogel‐based models have been shown to enhance drug resistance profiles, thereby offering insights into the mechanisms underlying therapeutic failures observed in clinical settings.^7^

Recent studies have highlighted the potential of hydrogel‐based 3D models in cervical cancer research, demonstrating their utility in drug screening and understanding tumor biology. For instance, multicellular tumor spheroids (MTSs) formed within hydrogels can recapitulate key features of in vivo tumors, including heterogeneity and resistance to chemotherapy agents.^8^ These models facilitate exploration of the TME's role in drug response and resistance, providing a platform for identifying novel therapeutic targets and strategies.^9^ Furthermore, the integration of immune cells and stromal components into these models allows for a more comprehensive understanding of the interactions that drive tumor progression and treatment outcomes.^10^, ^11^

**FIGURE 1:**
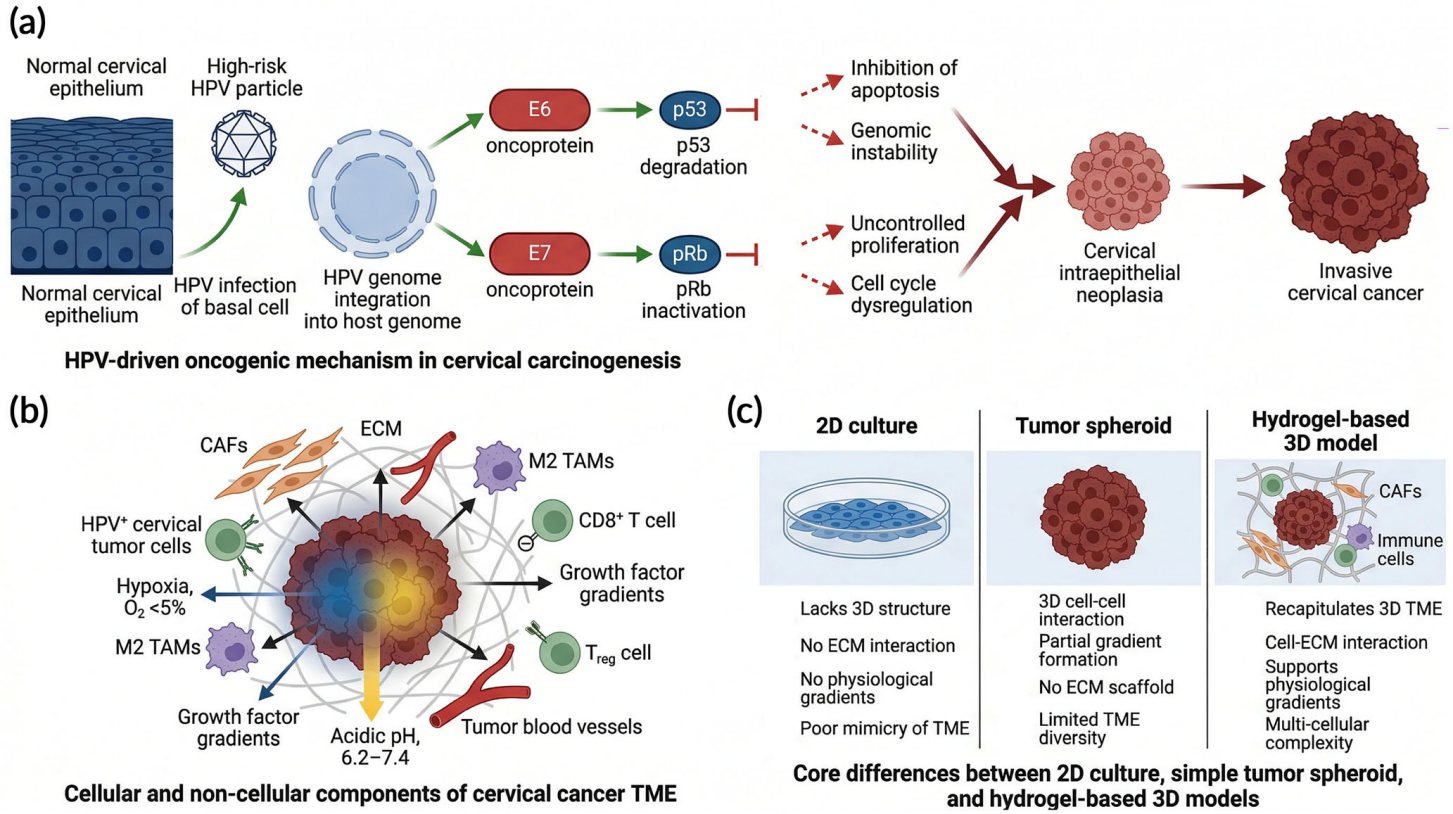
Core characteristics of HPV‐driven cervical cancer tumor microenvironment and limitations of traditional preclinical models. This figure systematically demonstrates the core oncogenic mechanism of high‐risk human papillomavirus (HPV)‐driven cervical tumorigenesis, the key compositional features of the cervical cancer tumor microenvironment (TME), and the core differences between distinct in vitro culture models, clarifying the irreplaceable value of hydrogel‐based 3D models in cervical cancer research.^2^, ^12^, ^13^, ^14^ (a) Illustrates the complete oncogenic pathway of high‐risk HPV infection driving malignant transformation of cervical epithelial cells. After high‐risk HPV particles infect the basal cells of normal cervical epithelium, the viral genome integrates into the host cell genome to drive the expression of E6 and E7 oncoproteins. Among them, E6 oncoprotein induces p53 protein degradation, thereby inhibiting cell apoptosis and promoting genomic instability; E7 oncoprotein inactivates pRb protein, resulting in cell cycle dysregulation and uncontrolled proliferation. These effects ultimately drive the occurrence of cervical intraepithelial neoplasia and its progression to invasive cervical cancer. (b) Displays the core cellular and non‐cellular components of the cervical cancer TME. The center is the HPV‐positive cervical tumor cell parenchyma, surrounded by a reticular structure of extracellular matrix (ECM). The core cellular components in the TME include cancer‐associated fibroblasts (CAFs), M2‐type tumor‐associated macrophages (M2 TAMs), exhausted CD8+ T cells, regulatory T cells (Treg), and tumor vascular endothelial cells. The non‐cellular features include the hypoxic microenvironment in the tumor core (oxygen concentration <5%), acidic pH gradient (pH 6.2–7.4), and growth factor gradients, fully presenting the spatial heterogeneity and functional characteristics of the cervical cancer TME. (c) Compares the core differences among three types of in vitro models for cervical cancer research, including 2D monolayer culture, simple multicellular tumor spheroid, and hydrogel‐based 3D model. It clarifies the core limitations of 2D monolayer culture, including the lack of 3D structure and ECM interaction, and the inability to recapitulate physiological gradients and TME characteristics. Although simple multicellular tumor spheroids can achieve 3D cell–cell interaction and basic gradient formation, they lack exogenous ECM scaffold and have limited capacity for TME component integration. In contrast, the hydrogel‐based 3D model can fully recapitulate the 3D TME characteristics of cervical cancer, realize cell–ECM interaction, and support the construction of physiological gradients and co‐culture of multi‐cellular components, which is a more physiologically relevant model for cervical cancer research. HPV, human papillomavirus; TME, tumor microenvironment; CAFs, cancer‐associated fibroblasts; TAMs, tumor‐associated macrophages; Treg, regulatory T cell; ECM, extracellular matrix.

In summary, the application of hydrogel‐based 3D tumor models represents a significant advancement in cancer research, particularly for cervical cancer. These models offer a more accurate representation of the TME, enabling researchers to investigate the complex interactions between cancer cells and their microenvironment. However, most current hydrogel‐based models remain phenomenological and descriptive. Critical limitations include insufficient mimicry of clinical TME complexity, lack of standardized protocols, and poor consistency with patient‐derived tissues. Compared with organoids or organ‐on‐chip systems, hydrogel models still face challenges in vascularization, immune component integration, and long‐term homeostasis. These gaps restrict their predictive value and clinical translation. This review aims to systematically summarize the latest applications of hydrogels in simulating the TME of cervical cancer and analyze their advantages and challenges in drug screening contexts (Figure 1).

## 2 BASIC PROPERTIES OF HYDROGELS AND THEIR ADVANTAGES

### 2.1 Physicochemical properties of hydrogels

Hydrogels are three‐dimensional (3D) polymeric networks capable of absorbing and retaining large amounts of water, often exceeding 90% by weight, which closely mimics the aqueous environment of biological tissues. This high water content, combined with their porous architecture, facilitates efficient diffusion of nutrients, oxygen, and metabolic waste products, thereby creating a microenvironment conducive to cell survival and function. The mechanical properties of hydrogels, such as elasticity modulus and hardness, are highly tunable through manipulation of polymer composition, crosslinking density, and fabrication methods. This tunability is crucial for simulating the biomechanical heterogeneity of cervical cancer tissues, which vary across different stages and grades of the disease. For example, hydrogels composed of polyethylene glycol (PEG) and poly(lactide glycolide) (PLGA) triblock copolymers exhibit thermo‐sensitive behavior with adjustable swelling properties and mechanical strength, enabling the sustained release of bioactive molecules like epidermal growth factor (EGF) to modulate tumor microenvironment stiffness and inhibit cervical cancer recurrence. In this case study, the authors optimized the polymer block ratio to achieve a sol–gel transition at physiological temperature (37°C), enabling minimally invasive in situ injection and gelation in the cervical surgical resection cavity of nude mice. The hydrogel exhibited tunable elastic modulus (ranging from 1.2 to 8.7 kPa) matching the mechanical heterogeneity of cervical tumor and adjacent normal tissues, with a sustained EGF release profile over 28 days in vitro. Through in vivo experiments using a HeLa cell‐derived xenograft mouse model, this hydrogel system was verified to reduce local tumor recurrence rate by 62.5% compared with the free EGF treatment group, via reversing matrix stiffness‐induced epithelial–mesenchymal transition (EMT) of residual cervical cancer cells. This case not only demonstrates the tunable physicochemical properties of thermo‐sensitive hydrogels for cervical cancer‐related applications, but also provides a validated strategy to link hydrogel mechanical regulation with tumor recurrence inhibition, which is a key design principle for hydrogel‐based cervical cancer models.^15^ Similarly, polysaccharide‐based hydrogels, such as alginate and chitosan composites, can be chemically crosslinked to achieve enhanced mechanical stability and controlled degradation rates, which are essential for long‐term cell culture and drug screening applications.^16^, ^17^ The degradation kinetics of hydrogels can be precisely controlled by adjusting crosslinker types and densities, allowing for sustained release of encapsulated therapeutics and maintaining scaffold integrity during extended in vitro or in vivo studies.^18^ Furthermore, advanced fabrication techniques, including 3D bioprinting and microfluidic‐assisted molding, enable the production of hydrogels with defined geometries and pore sizes, which influence cellular infiltration and tissue‐like organization.^19^, ^20^ Collectively, these physicochemical properties provide hydrogels with the versatility to closely replicate the complex mechanical and biochemical milieu of the cervical tumor microenvironment, thereby enhancing the physiological relevance of in vitro models for tumor biology and drug screening.

### 2.2 Biocompatibility of hydrogels and regulation of cell behavior

**FIGURE 2:**
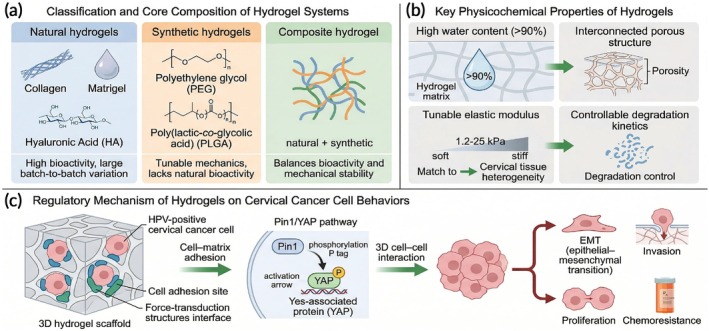
Core physicochemical/biological properties of hydrogels and their adaptability for cervical cancer modeling. This figure systematically demonstrates the classification system of hydrogel materials for cervical cancer 3D modeling, the core physicochemical properties of hydrogels adapted to cervical cancer research, and the core mechanism by which hydrogels regulate the malignant behaviors of cervical cancer cells through their biological characteristics. It fully clarifies the adaptation logic of hydrogels for in vitro 3D modeling of cervical cancer and provides theoretical support for the application of hydrogels in cervical cancer research.^12^, ^15^, ^18^, ^24^ (a) Shows the three major categories, core compositions, and corresponding advantages and limitations of hydrogel systems commonly used for cervical cancer 3D modeling. Including: (1) natural hydrogels, with core materials of collagen, Matrigel, and hyaluronic acid (HA). Their core advantage is high bioactivity to support cell adhesion and growth, while the limitation is large batch‐to‐batch variation and limited tunable range of mechanical properties. (2) Synthetic hydrogels, with core materials of polyethylene glycol (PEG) and poly(lactic‐co‐glycolic acid) (PLGA). Their core advantage is highly tunable mechanical properties and controllable degradation rate, while the limitation is the lack of natural bioactivity, requiring additional functional modification to support cell growth. (3) Composite hydrogels (natural + synthetic hybrid system), whose core advantage is balancing the bioactivity of natural materials and the mechanical stability of synthetic materials, which is the optimal material system for cervical cancer 3D modeling. (b) Displays the four core physicochemical properties of hydrogels adapted to cervical cancer 3D modeling. Including: (1) high water content (>90%), which highly mimics the aqueous microenvironment of biological tissues and supports efficient diffusion of nutrients and metabolic waste. (2) Interconnected porous structure, which provides physical space for cell infiltration, spatial arrangement and tissue‐like growth. (3) Tunable elastic modulus (1.2–25 kPa), which can accurately match the mechanical heterogeneity from normal cervical tissue, cervical intraepithelial neoplasia to invasive cervical cancer. (4) Controllable degradation kinetics, which can match the scaffold degradation rate with extracellular matrix deposition rate by adjusting crosslinking density, meeting the requirements of long‐term cell culture and sustained drug release. (c) Elucidates the regulatory mechanism of hydrogel biological properties on the malignant behaviors of cervical cancer cells. The 3D hydrogel scaffold mediates cell–matrix adhesion of HPV‐positive cervical cancer cells through cell adhesion sites and force–transduction structure interface, and then activates the Pin1/YAP core mechanotransduction pathway. Combined with 3D cell–cell interaction in the spatial structure, it ultimately regulates the core biological behaviors of cervical cancer cells, including epithelial–mesenchymal transition (EMT), invasion, proliferation, and chemoresistance, which clarifies the molecular mechanism of hydrogels simulating the pathophysiological process of cervical cancer. HA, hyaluronic acid; PEG, polyethylene glycol; PLGA, poly(lactic‐co‐glycolic acid); HPV, human papillomavirus; EMT, epithelial–mesenchymal transition; YAP, Yes‐associated protein.

The biocompatibility of hydrogels is a fundamental attribute that underpins their widespread use in biomedical applications, including 3D tumor modeling and drug delivery. Natural hydrogels derived from extracellular matrix (ECM) components such as collagen, gelatin, hyaluronic acid, and chitosan inherently provide bioactive motifs that facilitate specific cell–matrix interactions critical for cervical cancer cell adhesion, proliferation, and migration.^21^, ^22^ For instance, gelatin‐based hydrogels functionalized with RGD peptides enhance integrin‐mediated cell adhesion, promoting more physiologically relevant cellular behaviors.^23^ Synthetic hydrogels like PEG can be functionalized with bioactive ligands or peptides to precisely modulate cellular responses, offering a platform for tailored microenvironments that mimic native tumor ECM.^24^ The 3D architecture of hydrogels fosters cell–cell and cell–matrix interactions that are more representative of in vivo tumor tissues compared to 2D cultures. This spatial organization influences key cellular processes such as epithelial–mesenchymal transition (EMT), invasion, and drug resistance, which are pivotal in cervical cancer progression.^12^ The landmark case validated this point using collagen hydrogel systems with tunable stiffness (ranging from 1 to 25 kPa) to mimic the mechanical heterogeneity of normal cervical tissue, low‐grade cervical intraepithelial neoplasia, and invasive cervical cancer. In this study, the authors found that increased matrix stiffness significantly promoted EMT, migration, and invasion of HPV18‐positive HeLa and HPV16‐positive SiHa cervical cancer cells, and further identified the Pin1/YAP signaling axis as the core mechanotransduction pathway mediating this process, independent of the classical Hippo pathway. This case not only confirms that the tunable mechanical properties of hydrogels can faithfully simulate the stiffness gradient during cervical cancer progression, but also reveals that matrix stiffness is a key regulator of HPV‐positive cervical cancer cell malignant phenotype, which cannot be recapitulated in 2D cultures with fixed mechanical environment. Moreover, hydrogels can be engineered to possess dynamic mechanical properties, including viscoelasticity and tunable stiffness, which regulate mechanotransduction pathways in cancer cells and stromal components, further modulating tumor behavior and therapeutic responses. Moreover, hydrogels can be engineered to possess dynamic mechanical properties, including viscoelasticity and tunable stiffness, which regulate mechanotransduction pathways in cancer cells and stromal components, further modulating tumor behavior and therapeutic responses.^25^, ^26^ The incorporation of stimuli‐responsive elements into hydrogels enables controlled release of therapeutic agents and real‐time modulation of the tumor microenvironment, enhancing the precision of drug screening platforms.^27^, ^28^ Importantly, hydrogels have demonstrated excellent cytocompatibility with cervical cancer cell lines such as HeLa, supporting long‐term culture without inducing cytotoxic effects.^29^ Collectively, the intrinsic biocompatibility and customizable bioactivity of hydrogels provide a robust framework for regulating cervical cancer cell behavior in 3D culture systems, thereby improving the fidelity of tumor models for drug discovery and mechanistic studies (Figure 2).

## 3 KEY FEATURES OF THE 3D TUMOR MICROENVIRONMENT IN CERVICAL CANCER

### 3.1 Cellular composition and spatial architecture

The tumor microenvironment (TME) of cervical cancer is a complex and dynamic ecosystem shaped by HPV infection and distinctive stromal‐immune crosstalk, composed of various cellular constituents that interact intricately to influence tumor progression, invasion, and therapeutic response. Key cellular components within this microenvironment include malignant cervical tumor cells, cancer‐associated fibroblasts (CAFs) as core stromal regulators, immune cells such as exhausted T lymphocytes and tumor‐associated macrophages (the immune contexture of cervical cancer), and vascular endothelial cells forming the tumor vasculature. These diverse cell types contribute distinct biological functions and collectively shape the tumor's behavior. In three‐dimensional (3D) culture systems, cells exhibit self‐organizing capabilities to form organoid‐like structures that recapitulate the heterogeneity and spatial organization observed in vivo. This self‐organization enables the preservation of cell–cell and cell–matrix interactions critical for maintaining tumor architecture and function. The extracellular matrix (ECM), composed predominantly of proteins such as collagen and fibronectin, provides not only structural support but also biochemical cues that regulate tumor cell invasion and metastasis. The distribution and composition of ECM components influence tumor stiffness and porosity, thereby affecting cellular motility and interaction dynamics. Thus, understanding the cellular composition and spatial architecture in 3D models is essential for accurately mimicking the cervical cancer microenvironment and for developing effective drug screening platforms.^30^, ^31^, ^32^

### 3.2 Biochemical and mechanical signaling

The biochemical and mechanical milieu within the cervical cancer microenvironment plays a pivotal role in modulating HPV‐driven oncogenic signaling, tumor cell behavior, including epithelial–mesenchymal transition (EMT), invasion, and response to therapy. Biochemically, gradients of oxygen (hypoxia),^13^ pH,^14^ and growth factors^33^ are characteristic features of the tumor niche. The authors synthesized core‐shell silica‐based oxygen nanosensors and embedded them into collagen‐alginate hybrid hydrogels, which supported the 3D culture of HeLa cervical cancer spheroids. This system achieved real‐time live imaging of dissolved oxygen levels in the hydrogel constructs and successfully mapped the hypoxic core (oxygen concentration <5%) within 300 μm‐diameter HeLa spheroids, which was accompanied by significant upregulation of HIF‐1α and glycolysis‐related genes. In their subsequent 2025 work, they further developed modular pH‐sensing hybrid hydrogels, which achieved a pH detection range of 5.0–7.4 with high resolution, and accurately quantified the acidic extracellular pH (pH 6.2–6.5) in the core region of HeLa spheroids cultured in 3D hydrogels, consistent with the acidic TME observed in clinical cervical cancer specimens. These cases demonstrate that hydrogel systems can not only reconstruct the hypoxia and pH gradients characteristic of the cervical cancer TME but also integrate sensing functions to dynamically monitor these biochemical parameters, which is essential for studying tumor metabolism and drug efficacy under physiologically relevant conditions. Hypoxia, resulting from aberrant vascularization, induces stabilization of hypoxia‐inducible factors (HIFs), which orchestrate gene expression programs promoting angiogenesis, metabolic reprogramming, and immune evasion. pH gradients, often acidic in tumor cores due to anaerobic glycolysis, influence drug efficacy and cellular metabolism. Growth factor gradients, including vascular endothelial growth factor (VEGF) and transforming growth factor‐beta (TGF‐β), regulate angiogenesis and stromal cell activation. Mechanically, the stiffness of the ECM and the forces exerted by fluid flow within the tumor vasculature significantly impact tumor progression. Increased matrix stiffness has been shown to promote EMT by activating mechanotransduction pathways involving integrins and focal adhesion complexes, thereby enhancing tumor cell invasiveness and altering drug sensitivity. Furthermore, the crosstalk between mechanical and biochemical signaling pathways modulates immune cell function within the tumor microenvironment, affecting antitumor immunity. In cervical cancer 3D models, replicating these biochemical gradients and mechanical properties is crucial for faithful simulation of in vivo conditions and for evaluating therapeutic responses under physiologically relevant contexts.^34^, ^35^, ^36^, ^37^

### 3.3 Material selection and design

The selection and design of hydrogels for constructing three‐dimensional (3D) cervical cancer models are critical to accurately recapitulate the tumor microenvironment (TME) and enable reliable drug screening. Natural hydrogels, such as Matrigel, collagen, hyaluronic acid, and gelatin, possess inherent bioactivity due to their retention of extracellular matrix (ECM) proteins and signaling motifs, which support cell adhesion, proliferation, and differentiation. However, natural hydrogels often suffer from significant batch‐to‐batch variability and limited mechanical tunability, which can affect reproducibility and experimental consistency in 3D tumor modeling.^38^, ^39^ In contrast, synthetic hydrogels such as polyethylene glycol (PEG)‐based systems offer precise control over mechanical properties, degradation rates, and chemical functionalities. These materials allow for systematic tuning of stiffness and biochemical cues but lack intrinsic bioactivity, necessitating functionalization with cell‐adhesive peptides or ECM‐mimetic molecules to support cellular behaviors.^38^, ^40^ To harness the advantages of both natural and synthetic materials, composite hydrogels have been developed. For example, hybrid systems combining collagen, hyaluronic acid, and PEG have demonstrated improved mechanical stability while maintaining biological relevance, enabling more faithful modeling of the cervical cancer ECM.^39^, ^41^ Furthermore, advancements in bioink formulations have propelled the development of 3D bioprinting techniques, which facilitate the fabrication of cervical cancer models with spatial precision and heterogeneity. Bioinks composed of natural polymers (e.g., gelatin methacryloyl, alginate) combined with synthetic components allow for tailored rheological properties suitable for extrusion bioprinting, supporting high cell viability and complex tissue architectures.^42^, ^43^, ^44^ Dynamic bioinks incorporating supramolecular or reversible covalent crosslinks provide additional benefits such as shear‐thinning behavior and self‐healing, enhancing printability and mimicking the dynamic nature of the tumor ECM.^44^ The integration of nanocomposite materials into bioinks further improves mechanical strength and biological functionality, crucial for replicating the cervical tumor microenvironment.^45^ Collectively, the strategic selection and design of hydrogel materials—balancing bioactivity, mechanical properties, and printability—are foundational for constructing robust 3D cervical cancer models that enable physiologically relevant studies and effective drug screening.(Tables 1 and 2) Compared with simple spheroid models, hydrogels provide a physiological ECM environment but suffer from higher variability and poorer reproducibility. Compared with organ‐on‐chip platforms, hydrogel systems lack real‐time fluidic control and spatial gradient precision. Natural hydrogels offer high bioactivity but poor batch consistency; synthetic hydrogels provide uniformity but insufficient biological cues. This trade‐off remains a key limitation.

**TABLE 1:** Comparison of hydrogel materials for cervical cancer 3D models.

Hydrogel type	Representative compositions	Core advantages	Typical applications
Natural hydrogels	Matrigel, collagen, hyaluronic acid, gelatin	Inherent bioactivity; supports native cell behaviors	Basic TME simulation; tumor organoid formation
Synthetic hydrogels	Polyethylene glycol (PEG)‐based polymers	Precise mechanical control; stable degradation; uniform batch‐to‐batch quality	Functionalized models for targeted microenvironment regulation
Composite hydrogels	Collagen‐PEG, alginate‐gelatin, nanocomposite bioinks	Balances bioactivity and mechanical stability; printability	3D bioprinting of heterogeneous TME; long‐term drug screening
Dynamic bioinks	Gelatin methacryloyl + synthetic crosslinkers; supramolecular networks	Shear‐thinning, self‐healing properties; dynamic stiffness modulation	Fabrication of complex, adaptive tumor architectures

**TABLE 2:** Core strategies and technologies for hydrogel‐based 3D cervical cancer TME construction.

Modeling strategy	Key technologies	Regulatory parameters	Simulated TME characteristics
Static 3D culture	Hydrogel encapsulation, tumor spheroid formation	Cell density, hydrogel stiffness, culture duration	Basic cell–matrix interaction, tumor spheroid heterogeneity
Gradient microenvironment construction	Photocrosslinking regulation, microfluidic gradient generation	Stiffness gradient, oxygen gradient, drug gradient	Hypoxic regions, pH gradient, matrix stiffness heterogeneity
Multicellular co‐culture	Tumor cell–fibroblast–immune cell co‐culture	Cell ratio, seeding method	Tumor‐stroma interaction, immune evasion, angiogenesis
Dynamic perfusion culture	Perfusion bioreactor, microfluidic chamber	Fluid shear stress, mass exchange rate	Tumor interstitial flow, intravascular blood flow, nutrient transport
3D bioprinting	Extrusion printing, photopolymerization printing	Scaffold structure, spatial arrangement, pore size	Tumor spatial heterogeneity, tissue structure bionics

### 3.4 Microenvironment parameter regulation techniques

**FIGURE 3:**
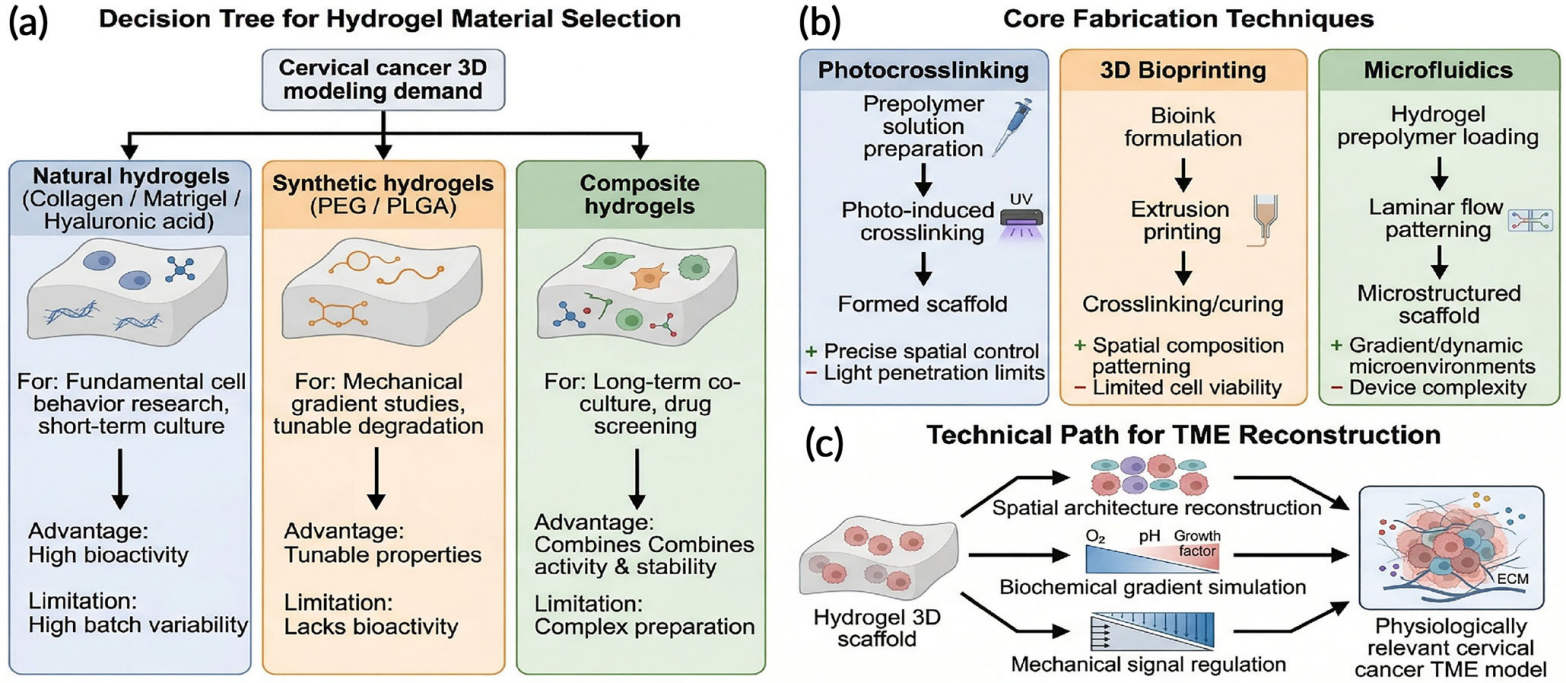
Hydrogel material design and technical system for 3D cervical cancer tumor microenvironment modeling. This figure systematically demonstrates the full‐chain technical system for the construction of hydrogel‐based 3D cervical cancer models, including the decision‐making logic of hydrogel material selection based on cervical cancer modeling requirements, the workflow of core model fabrication techniques, and the technical path for reconstructing the core features of cervical cancer tumor microenvironment (TME) using hydrogel models. It provides a systematic technical framework for the standardized construction and application of in vitro 3D cervical cancer models.^44^, ^46^, ^48^ (a) Illustrates the decision tree for hydrogel material selection based on cervical cancer 3D modeling requirements. It is divided into three major material branches: (1) natural hydrogels, with core materials of collagen, Matrigel, and hyaluronic acid, which are suitable for fundamental cell behavior research and short‐term in vitro culture. Their core advantage is high bioactivity, while the limitation is large batch‐to‐batch variability. (2) Synthetic hydrogels, with core materials of polyethylene glycol (PEG) and poly(lactic‐co‐glycolic acid) (PLGA), which are suitable for mechanical gradient studies and tunable degradation‐related experiments. Their core advantage is highly tunable physicochemical properties, while the limitation is the lack of natural bioactivity. (3) Composite hydrogels (natural + synthetic hybrid system), which are suitable for long‐term multicellular co‐culture and drug screening research. Their core advantage is balancing the bioactivity of natural materials and the mechanical stability of synthetic materials, while the limitation is a relatively complex preparation process. (b) Displays the complete workflow, core advantages and limitations of three major fabrication techniques for hydrogel‐based 3D cervical cancer models. Including: (1) photocrosslinking technique involves prepolymer solution preparation, UV‐induced crosslinking, and scaffold formation. Its core advantage lies in the precise spatial control of crosslinking density and stiffness, while its key limitation is the restricted light penetration depth. (2) 3D bioprinting technique involves bioink formulation, extrusion printing, and crosslinking/curing. Its core advantage is the precise spatial patterning of model composition and structure, while a key limitation is the compromised cell viability during the printing process. (3) The microfluidics technique involves hydrogel prepolymer loading, laminar flow patterning, and microstructured scaffold formation. It enables the construction of gradient and dynamic in vitro microenvironments as its core advantage, yet is constrained by the high complexity of device design and operation. (c) Elucidates the core technical path for reconstructing physiologically relevant cervical cancer TME via hydrogel models. Based on the hydrogel 3D scaffold, the accurate reconstruction of core TME features is achieved through three parallel technical paths: spatial architecture reconstruction via co‐culture of tumor cells, stromal and immune cells; biochemical microenvironment simulation via mimicking the gradient distribution of oxygen, pH and growth factors; mechanical signal regulation via tuning scaffold stiffness. These paths ultimately converge to establish a physiologically relevant in vitro model of cervical cancer TME. TME, tumor microenvironment; PEG, polyethylene glycol; PLGA, poly(lactic‐co‐glycolic acid); UV, ultraviolet; ECM, extracellular matrix.

Replicating the complex and heterogeneous tumor microenvironment (TME) of cervical cancer within 3D hydrogel models necessitates advanced techniques to spatially and temporally regulate microenvironmental parameters. Photocrosslinking methods, such as UV or visible light‐induced polymerization, enable precise spatial control of hydrogel stiffness and crosslink density, thereby creating mechanical gradients that mimic the stiffness heterogeneity observed in tumors. This spatial heterogeneity influences cancer cell behavior, including migration and drug response.^38^, ^46^ Microfluidic technologies further enhance microenvironmental control by generating biochemical gradients of oxygen, nutrients, and drugs within hydrogel constructs. Gradient generators employing laminar flow or diffusion‐based designs can establish stable and tunable gradients, allowing simulation of hypoxic zones and drug concentration variations characteristic of cervical tumors.^47^, ^48^ For example, microfluidic chips with embedded microchambers and ECM composites have been used to model nutrient and drug gradients, facilitating studies on tumor cell proliferation and migration under physiologically relevant conditions.^49^ Moreover, microfluidic mixing probes and inertial microfluidic devices have been developed to produce multiple discrete concentration gradients efficiently, enabling high‐throughput drug screening with precise dosage control.^50^, ^51^ Dynamic culture systems, such as perfusion bioreactors and microfluidic flow chambers, simulate the in vivo fluidic environment by providing continuous medium flow and shear stress, which are critical for maintaining cell viability and function, as well as for mimicking interstitial flow in tumors.^48^, ^52^ These systems facilitate nutrient and waste exchange, enhance mass transport, and replicate mechanical stimuli, thereby improving the physiological relevance of 3D cervical cancer models. Additionally, advanced fabrication techniques inspired by movable type printing and modular microfluidics allow rapid prototyping of customizable microenvironments with integrated flow control and gradient generation capabilities.^53^ Overall, the integration of photocrosslinking, microfluidic gradient generation, and dynamic perfusion culture represents a powerful approach to engineer spatially heterogeneous and dynamically regulated 3D cervical cancer microenvironments, which are indispensable for accurate tumor modeling and effective drug screening (Figure 3).

## 4 APPLICATION OF HYDROGEL MODELS IN DRUG SCREENING FOR CERVICAL CANCER

### 4.1 Chemotherapy drug sensitivity testing

Chemotherapy remains a cornerstone in cervical cancer treatment, yet traditional two‐dimensional (2D) cell culture models often fail to accurately predict clinical drug responses due to their inability to replicate the complex tumor microenvironment (TME) and HPV‐related drug resistance mechanisms. Hydrogel‐based three‐dimensional (3D) culture models have emerged as superior platforms that mimic the extracellular matrix (ECM) and physical properties of cervical tumors, thus providing more clinically relevant drug sensitivity data. Studies have demonstrated that cervical cancer cells cultured in 3D hydrogels exhibit differential sensitivity to chemotherapeutic agents such as cisplatin compared to 2D cultures, with 3D models better recapitulating the chemoresistance observed in patients.^54^ This difference is partly attributed to the hydrogels' ability to simulate the tumor stroma's mechanical barriers and interstitial pressure, which affect drug penetration and efficacy. For instance, hydrogels constructed from polyglycidol/polyacrylamide matrices have shown enhanced delivery and selective cytotoxicity of 5‐fluorouracil (5‐FU) to HeLa cervical cancer cells, while sparing normal cells, indicating improved drug selectivity in 3D systems. In this work, the authors fabricated a hydrogel with a porous interconnected structure (pore size 50–150 μm) and tunable stiffness, which supported the long‐term 3D culture of HeLa cells and the formation of multicellular tumor spheroids (MTSs) with a diameter of 100–300 μm, closely mimicking the in vivo tumor nodule structure. Through 14 days of drug exposure experiments, the study found that 5‐FU loaded in this hydrogel system exhibited a 4.8‐fold higher selective cytotoxicity to HeLa cells in 3D culture compared with 2D monolayer culture, with a half‐maximal inhibitory concentration (IC50) of 12.7 μM in 3D versus 2.6 μM in 2D. Critically, this hydrogel system significantly reduced the toxic effect of 5‐FU on human normal cervical epithelial cells, with cell viability remaining above 85% at the therapeutic concentration. This case directly validates that hydrogel‐based 3D models can better recapitulate the clinical chemoresistance and drug penetration barriers of cervical tumors, and provides a quantitative reference for the dose setting of preclinical chemotherapy drug screening.^54^ Furthermore, hydrogels allow for the assessment of drug penetration by mimicking the ECM density and composition, which is crucial for predicting in vivo drug bioavailability and therapeutic outcomes. Long‐term culture of cervical cancer cells within hydrogels also facilitates the study of acquired chemoresistance mechanisms, as cells adapt to the 3D microenvironment and drug exposure over time.^21^ Collectively, hydrogel‐based 3D culture systems provide a physiologically relevant platform for chemotherapy drug sensitivity testing in cervical cancer, offering improved prediction of clinical responses and insights into resistance development (Table 3).

**TABLE 3:** Biological readouts of hydrogel models in cervical cancer research.

Assay type	Core biological readouts	Detection methods	Application purpose
Cell behavior assay	Proliferation, apoptosis, invasion, migration, EMT	Live/dead staining, Transwell, scratch test, immunofluorescence	Evaluate malignant phenotypes of tumor cells
Microenvironment characterization	Hypoxia, matrix stiffness, angiogenesis	Hypoxia probe, atomic force microscopy, tube formation assay	Simulate and verify key TME features
Drug response assay	Drug sensitivity, drug resistance, drug penetration	CCK‐8, flow cytometry, fluorescence tracing	Chemotherapy/targeted/immunotherapy screening
Omics and molecular detection	Transcriptome, metabolome, protein expression	Single‐cell sequencing, metabolic flux analysis, Western blot	Reveal molecular mechanisms and drug resistance pathways

### 4.2 Evaluation of targeted therapy and immunotherapy

**FIGURE 4:**
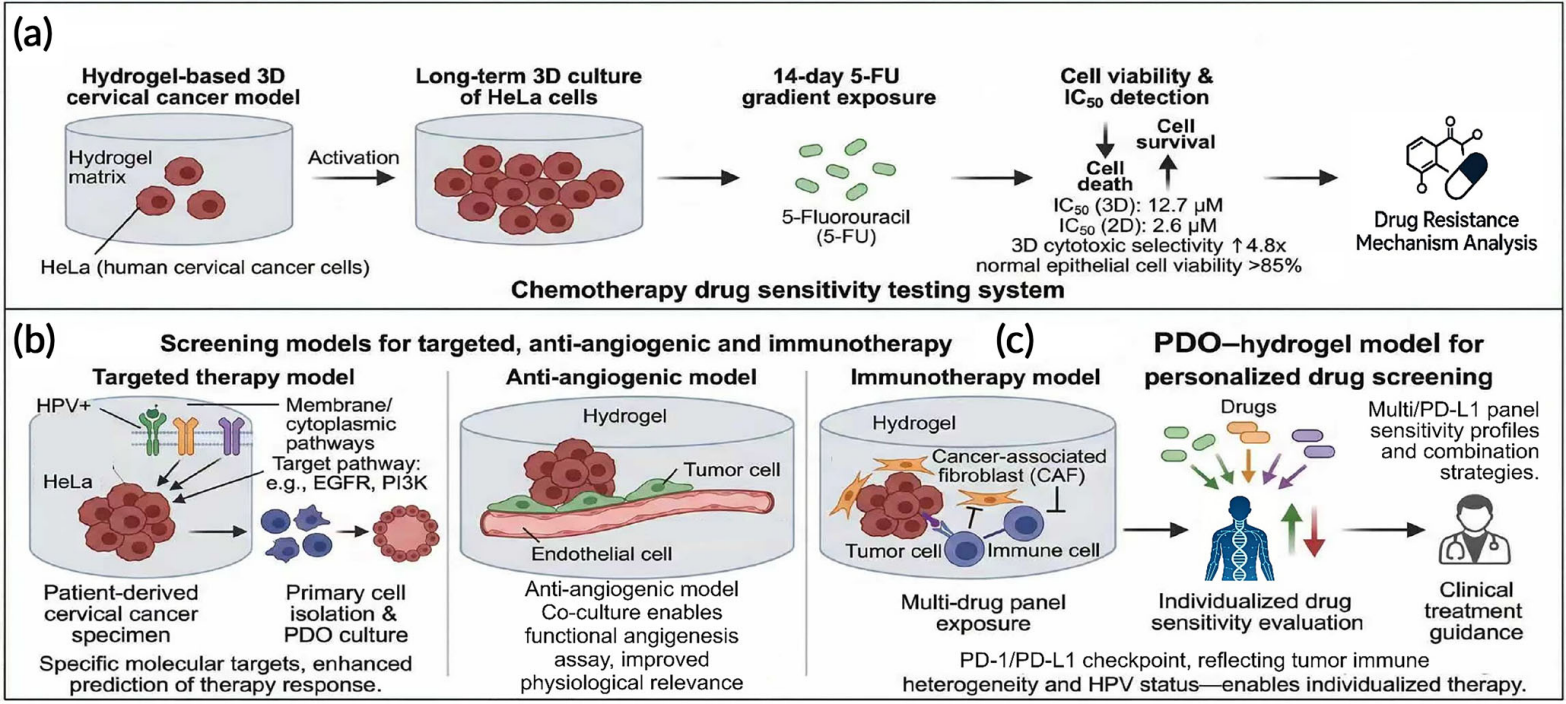
Application progress of hydrogel‐based 3D models in cervical cancer drug screening and personalized therapy. This figure systematically demonstrates the complete application system of hydrogel‐based 3D cervical cancer models in chemotherapy drug sensitivity testing, targeted/anti‐angiogenic/immunotherapy screening, and patient‐derived organoid (PDO)‐based personalized drug screening. It intuitively presents the core design, key quantitative results, and clinical translational value of different screening models, providing a visualized technical framework for preclinical drug evaluation and personalized therapy of cervical cancer.^54^, ^55^, ^56^ (a) Illustrates the complete workflow and core research conclusions of chemotherapy drug sensitivity testing based on hydrogel‐based 3D models. A hydrogel‐based 3D cervical cancer model was constructed using HeLa cells. After long‐term 3D culture, 14‐day gradient exposure to 5‐fluorouracil (5‐FU) was performed. Cell viability and half‐maximal inhibitory concentration (IC₅₀) detection revealed that the IC₅₀ of 5‐FU against HeLa cells in the 3D model was 12.7 μM, significantly higher than 2.6 μM in 2D culture. The 3D system exhibited a 4.8‐fold increase in selective cytotoxicity to cervical cancer cells compared with 2D culture, and the viability of normal cervical epithelial cells remained above 85% at therapeutic concentrations, verifying that hydrogel‐based 3D models can better recapitulate the clinical chemoresistance characteristics of cervical cancer. (b) Displays the design of three hydrogel‐based screening models for non‐chemotherapy: (1) Targeted therapy model: PDO hydrogel models constructed from patient‐derived cervical cancer specimens can precisely target molecular pathways such as EGFR and PI3K, improving the prediction accuracy of therapeutic response. (2) Anti‐angiogenic model: Co‐culture of tumor cells and vascular endothelial cells in hydrogel enables functional angiogenesis assay, enhancing the physiological relevance of the model. (3) Immunotherapy model: Co‐culture of tumor cells, cancer‐associated fibroblasts (CAFs), and immune cells in hydrogel can simulate the inhibitory effect of PD‐1/PD‐L1 immune checkpoints, reflecting tumor immune heterogeneity and HPV status, supporting the evaluation of immunotherapy efficacy. (c) Elucidates the complete application workflow of PDO‐hydrogel based personalized drug screening. Primary cells were isolated from patient cervical cancer specimens and used to construct PDOs, which were then embedded in hydrogel scaffolds. Multi‐drug panel exposure and individualized drug sensitivity evaluation were performed to generate multi‐drug/PD‐L1 sensitivity profiles and combination therapy strategies, providing precise guidance for clinical treatment. This model fully retains the genetic heterogeneity and HPV status of the patient's tumor, serving as a core tool for realizing personalized cervical cancer therapy. HeLa, human cervical cancer cell line; 5‐FU, 5‐Fluorouracil; IC₅₀, half‐maximal inhibitory concentration; PDO, patient‐derived organoid; CAFs, cancer‐associated fibroblasts; EGFR, epidermal growth factor receptor; PI3K, phosphatidylinositol 3‐kinase; PD‐1/PD‐L1, programmed cell death protein 1/ligand 1.

Hydrogel models have also been instrumental in evaluating targeted therapies and immunotherapies for cervical cancer by recapitulating the HPV‐associated immune contexture and stromal interactions, enabling co‐culture of tumor and immune cells. The ability of hydrogels to support 3D co‐cultures facilitates the assessment of immune checkpoint inhibitors, such as PD‐1/PD‐L1 blockade, by providing a controlled environment to study HPV‐related tumor‐immune suppression and stromal crosstalk.^27^ Additionally, hydrogel‐based vascularized tumor models have been developed to screen anti‐angiogenic agents like bevacizumab, which target tumor blood vessel formation critical for cervical cancer progression.^55^ Patient‐derived organoid (PDO) models embedded within hydrogels represent a significant advancement in personalized medicine, as they maintain the genetic and phenotypic heterogeneity of individual tumors and their microenvironment including HPV status and immune‐stromal features.^56^ PDOs have been successfully used to perform individualized drug sensitivity testing, including targeted agents and immunotherapies, thereby guiding tailored treatment regimens for cervical cancer patients. The integration of hydrogels with advanced technologies such as 3D bioprinting and microfluidics further enhances the complexity and throughput of these models, enabling high‐throughput screening of combinatorial therapies and real‐time monitoring of tumor responses.^20^ Overall, hydrogel‐based models provide versatile and physiologically relevant platforms to evaluate the efficacy of targeted therapies and immunotherapies in cervical cancer, supporting the development of precision oncology approaches (Table 4 and Figure 4).

**TABLE 4:** Typical applications of hydrogel models in cervical cancer drug screening.

Drug category	Hydrogel type	Core model	Key research findings
Chemotherapeutic agents	Polyglycidol/polyacrylamide, PEG‐based hydrogels	3D cell spheroids, hypoxic models	Better recapitulate clinical drug resistance; improve selectivity and efficacy prediction of 5‐fluorouracil and cisplatin
Targeted agents	Vascularized composite hydrogels	Vascularized tumor‐on‐a‐chip	Enable screening of anti‐angiogenic drugs (bevacizumab); evaluate drug effects on tumor blood vessels
Immunotherapeutic agents	Functionalized gelatin/hyaluronic acid hydrogels	Tumor‐immune cell co‐culture models	Allow evaluation of PD‐1/PD‐L1 inhibitors; simulate tumor immune evasion and immune cell cytotoxicity
Personalized therapy	Patient‐derived organoid (PDO) composite hydrogels	Patient‐derived tumor organoids	Preserve patient tumor heterogeneity; achieve individualized drug sensitivity testing

## 5 CURRENT CHALLENGES AND LIMITATIONS OF HYDROGEL‐BASED 3D CERVICAL CANCER MODELS

### 5.1 Model standardization and validation

Major unresolved challenges include the lack of standardized protocols for hydrogel composition, stiffness, and cell seeding density, which severely compromises cross‐laboratory reproducibility. Most hydrogel‐based models remain structurally incomplete due to the absence of functional vasculature, lymphatics, neurons, and fully integrated adaptive immune components. A further critical limitation is the clinical disconnection, as few platforms faithfully recapitulate patient‐specific genomic heterogeneity and clinical drug responses. Compared with alternative models, hydrogels are less scalable than 2D cultures, less structurally stable than organoids, and less physiologically dynamic than organ‐on‐chip systems. In addition, functional immaturity persists as long‐term culture, stable perfusion, and immune homeostasis remain difficult to maintain. The establishment of standardized models and rigorous validation protocols remains a critical challenge in the development and application of hydrogel‐based three‐dimensional (3D) tumor microenvironment (TME) models for cervical cancer research. A significant obstacle is the lack of unified culture parameters and quality control standards, which adversely affects the reproducibility and comparability of experimental outcomes across different laboratories and studies. Without consensus on key variables such as hydrogel composition, stiffness, cell seeding density, and culture duration, the biological relevance and translational potential of these 3D models are compromised. Furthermore, the correlation between in vitro 3D hydrogel models and clinical specimens is often insufficiently validated. The scarcity of patient‐derived models, including primary tumor cells embedded within hydrogels that recapitulate the native extracellular matrix (ECM), limits the ability to faithfully mimic the heterogeneity and complexity of the cervical cancer microenvironment. This gap underscores the need to establish additional patient‐derived xenograft (PDX) or organoid‐based hydrogel models that can better represent the clinical scenario. Collectively, advancing model standardization and validation through consensus protocols, incorporation of clinically relevant patient‐derived materials, and development of HTS‐compatible platforms will be pivotal for translating hydrogel‐based 3D cervical cancer models into reliable tools for drug discovery and personalized medicine.^57^, ^58^, ^59^

### 5.2 Multi‐omics integrated analysis

The integration of multi‐omics technologies with hydrogel‐based 3D cervical cancer models represents a transformative approach to dissect the cellular heterogeneity and dynamic metabolic reprogramming within the tumor microenvironment. Single‐cell sequencing technologies, particularly single‐cell RNA sequencing (scRNA‐seq), have been instrumental in resolving the complex cellular compositions and transcriptional states of cells cultured in 3D hydrogels, enabling the identification of distinct tumor and stromal subpopulations and their interactions. Metabolomics and metabolic flux analyses further complement these data by revealing metabolic adaptations and pathway alterations induced by drug treatments or microenvironmental cues within the hydrogel matrices. Additionally, advanced imaging modalities such as light‐sheet fluorescence microscopy facilitate real‐time, dynamic visualization of 3D tumor spheroids or hydrogel‐embedded cultures, enabling longitudinal assessment of cellular behaviors, drug penetration, and microenvironmental changes without disrupting the 3D architecture. However, challenges remain in harmonizing data types, managing high‐dimensional datasets, and ensuring biological interpretability. Continued development of standardized protocols for multi‐omics data acquisition from hydrogel‐based models and advanced bioinformatics tools will be essential to fully exploit their potential in cervical cancer research and drug screening.^60^, ^61^, ^62^, ^63^, ^64^

## 6 FUTURE PERSPECTIVES AND ADVANCED DEVELOPMENT DIRECTIONS

### 6.1 Multi‐organ chip systems

The integration of multi‐organ chip systems represents a promising future direction for enhancing the physiological relevance of in vitro cervical cancer models, particularly in simulating systemic drug efficacy and toxicity. By combining cervical cancer tissue models with other organotypic cultures such as liver and vascular tissues, these platforms enable the evaluation of whole‐body pharmacokinetics and pharmacodynamics, which are crucial for accurate drug screening and safety assessment. Multi‐organ chips employ microfluidic technologies to facilitate controlled fluidic communication between discrete tissue compartments, thereby mimicking inter‐organ crosstalk mediated by soluble factors, extracellular vesicles, and cellular interactions. For instance, systems like the TissUse HUMIMIC Starter have demonstrated the ability to model interactions between gut, liver, brain, and kidney tissues, offering insights into metabolic and inflammatory processes relevant to drug metabolism and toxicity. In this study, the authors established a coupled platform of human liver equivalents and multi‐organ tissues (gut, brain, kidney) via microfluidic perfusion, with a continuous dynamic culture cycle of up to 28 days while maintaining tissue viability and function. Using this system, the team quantitatively evaluated the hepatic metabolism and off‐target toxicity of 12 clinically commonly used anti‐tumor drugs and found that the 4‐organ chip system could accurately predict the clinical hepatotoxicity and renal toxicity of drugs with a consistency of over 82%, which was significantly higher than that of traditional 2D static culture (37%). For cervical cancer research, this case provides a mature technical paradigm: by coupling hydrogel‐based cervical tumor models with liver, kidney, and other organ equivalents on a multi‐organ chip, it is possible to simultaneously evaluate the anti‐tumor efficacy, hepatic metabolic activation, and systemic toxicity of drug candidates in a single platform, which is the key to bridging the gap between preclinical testing and clinical outcomes.^65^ The incorporation of liver equivalents is particularly vital for cervical cancer drug screening, as hepatic metabolism significantly influences drug bioavailability and systemic effects. Microfluidic platforms enable dynamic exchange of nutrients, metabolites, and signaling molecules, replicating the physiological milieu more faithfully than static cultures.^66^ Furthermore, advanced multi‐organ systems are equipped with dynamic monitoring capabilities, including integrated biosensors and imaging modules, to track drug metabolism and pharmacokinetics in real time.^67^ These features allow for longitudinal assessment of drug responses, capturing both immediate and delayed effects on tumor and non‐tumor tissues. Recent developments also include robotic fluidic coupling and automated culture systems that maintain viability and function of multiple organ chips over extended periods, enhancing reproducibility and throughput.^68^ Despite technological challenges such as ensuring biological relevance, sensor integration, and platform interoperability, multi‐organ chip systems hold substantial potential to bridge the translational gap between preclinical models and clinical outcomes in cervical cancer therapeutics.^69^ By enabling systemic evaluation within a controlled microphysiological context, these platforms can facilitate the identification of efficacious and safe drug candidates, ultimately accelerating personalized medicine approaches for cervical cancer patients.

### 6.2 Intelligent responsive hydrogels

**FIGURE 5:**
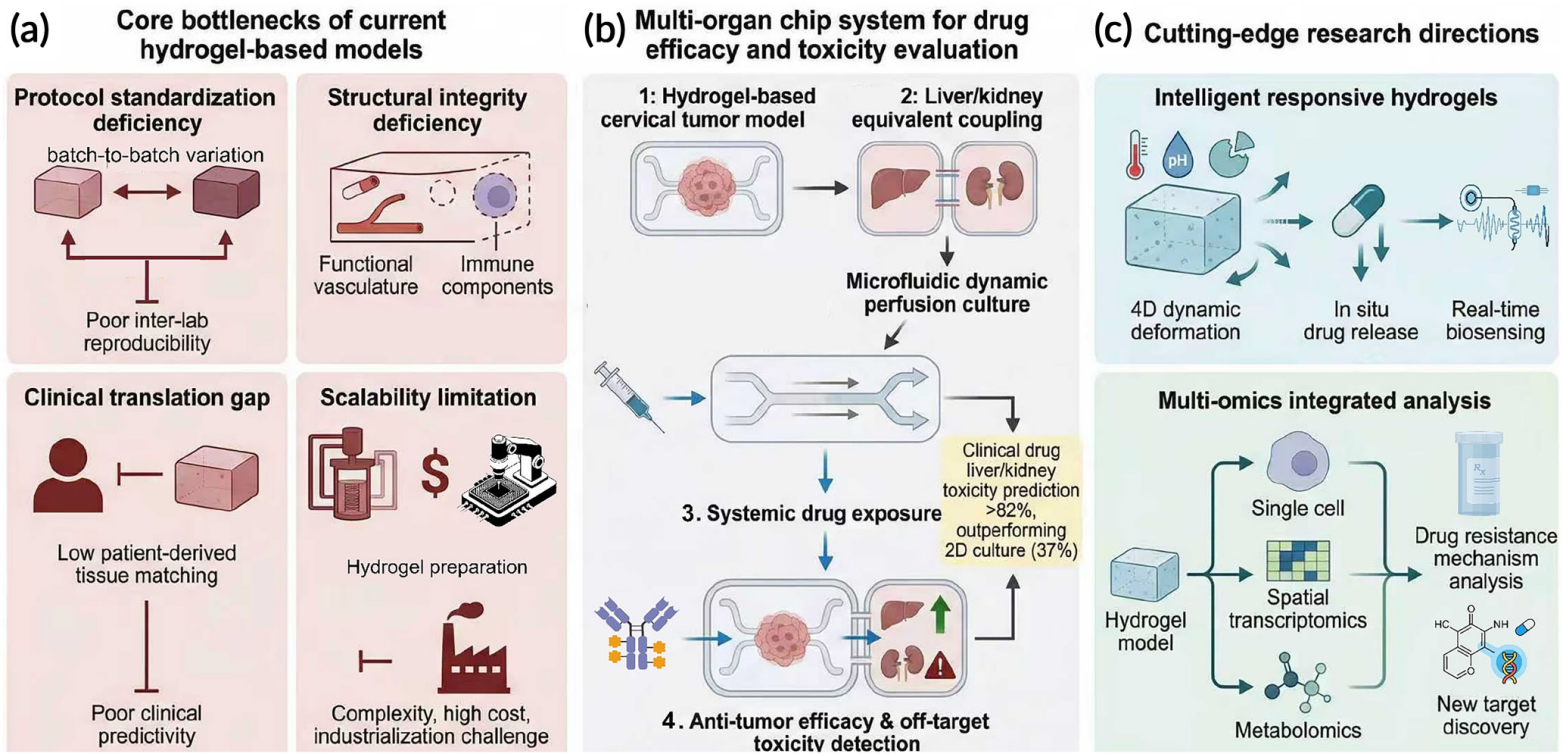
Current bottlenecks and future development directions of hydrogel‐based 3D cervical cancer models. This figure systematically summarizes the core bottlenecks of current hydrogel‐based 3D cervical cancer models, demonstrates the drug evaluation workflow and core performance of multi‐organ chip systems, and prospects two cutting‐edge research directions: intelligent responsive hydrogels and multi‐omics integrated analysis, providing critical analysis and forward‐looking guidance for technological breakthroughs and clinical translation in this field.^65^, ^70^, ^74^ (a) Highlights four core bottlenecks of current hydrogel‐based 3D cervical cancer models: (1) deficiency in protocol standardization, characterized by large batch‐to‐batch variation and poor inter‐laboratory reproducibility; (2) deficiency in structural integrity, lacking functional vascular networks and immune components to fully simulate the physiological TME; (3) clinical translation gap, with low patient‐derived tissue matching and poor clinical predictivity; (4) scalability limitation, featuring high complexity and cost of preparation and chip devices, hindering industrialization. (b) Illustrates the complete workflow of drug efficacy and toxicity evaluation using a multi‐organ chip system. The workflow includes: (1) construction of a hydrogel‐based cervical cancer tumor model; (2) coupling of liver/kidney organ equivalents with microfluidic dynamic perfusion culture; (3) systemic drug exposure; (4) simultaneous detection of anti‐tumor efficacy and off‐target toxicity. This system achieves >82% consistency in predicting clinical drug hepatotoxicity and nephrotoxicity, significantly outperforming 2D culture (37%), enabling concurrent evaluation of drug efficacy, metabolic activation, and systemic toxicity. (c) Prospects two cutting‐edge research directions: (1) intelligent responsive hydrogels, which enable pH/temperature/enzyme‐responsive 4D dynamic deformation, in situ controlled drug release, and real‐time biosensing, providing technical support for constructing dynamic physiological microenvironments; (2) multi‐omics integrated analysis, which combines hydrogel models with single‐cell sequencing, spatial transcriptomics, and metabolomics to dissect drug resistance mechanisms and discover novel therapeutic targets. TME, tumor microenvironment; PDO, patient‐derived organoid.

The development of intelligent responsive hydrogels constitutes a cutting‐edge approach to replicating the dynamic and heterogeneous tumor microenvironment of cervical cancer, offering enhanced capabilities for modeling pathological stimuli and monitoring cellular responses. These smart hydrogels are engineered to respond to specific internal stimuli such as pH shifts, enzymatic activity, or temperature variations, which are characteristic of tumor progression and therapeutic interventions. For example, pH‐sensitive hydrogels can mimic the acidic tumor microenvironment, enabling controlled drug release or structural changes in response to local acidity.^70^ Enzyme‐responsive hydrogels exploit overexpressed proteases within tumors to trigger degradation or cargo release, providing spatial and temporal precision in drug delivery.^71^ Temperature‐sensitive hydrogels, such as those based on methylcellulose or poly(N‐isopropylacrylamide), undergo sol–gel transitions near physiological temperatures, facilitating minimally invasive administration and in situ gelation.^72^ Integration of biosensors within these hydrogels enables real‐time monitoring of cellular behaviors and drug effects, employing optical, electrochemical, or impedance‐based detection modalities.^73^ Moreover, the advent of 4D hydrogels introduces the dimension of time‐dependent changes in hydrogel properties, enabling the simulation of disease progression and evolving microenvironmental conditions.^74^ The combination of multi‐stimuli responsiveness and biosensing capabilities positions intelligent hydrogels as versatile platforms for personalized drug screening and mechanistic studies in cervical cancer. Challenges remain in optimizing mechanical strength, biocompatibility, and response specificity, but advances in interpenetrating polymer networks and nanocomposite formulations are addressing these issues.^75^ Overall, intelligent responsive hydrogels represent a transformative technology for creating adaptive, biomimetic tumor models that enhance the predictive power of preclinical testing and facilitate the development of more effective, targeted therapies for cervical cancer (Figure 5).

## 7 CONCLUSION

In conclusion, hydrogels have emerged as a highly biomimetic and versatile platform for simulating the three‐dimensional tumor microenvironment of cervical cancer, significantly enhancing the predictive accuracy of drug screening processes. By enabling precise modulation of physicochemical properties and bioactivity, these hydrogels allow for the construction of cervical cancer models that faithfully recapitulate various disease stages, as well as the HPV‐driven biology, immune contexture, and stromal interactions unique to cervical cancer, thereby providing a more relevant context for studying tumor biology and therapeutic responses.

The impact of hydrogel‐based 3D models extends across multiple therapeutic modalities, including chemotherapy, targeted therapy, and immunotherapy. Their unique ability to mimic the complex extracellular matrix and cellular interactions within the tumor microenvironment offers unparalleled advantages in evaluating drug efficacy and resistance mechanisms.

Balancing the diverse research perspectives and findings in this field requires a critical appraisal of both the strengths and limitations inherent to hydrogel technologies. While these models provide superior physiological relevance compared to conventional systems, challenges remain in standardizing hydrogel formulations and culture conditions to ensure reproducibility and comparability across studies. Moreover, enhancing the clinical relevance of these models demands integration with patient‐derived cells and validation against clinical outcomes.

Looking forward, the future of hydrogel‐based cervical cancer modeling lies in the incorporation of intelligent, stimuli‐responsive hydrogels and the integration with multi‐organ‐on‐chip platforms. Such innovations promise to capture dynamic tumor‐host interactions and systemic responses, thereby refining the predictive power of preclinical models.

In summary, hydrogel platforms represent a transformative tool in cervical cancer research, offering a sophisticated means to simulate the tumor microenvironment with high fidelity. By addressing current challenges and embracing emerging technologies, these models hold the potential to revolutionize drug development and precision oncology, ushering in a new era of targeted and effective therapies for cervical cancer patients.

**FIGURE 6:**
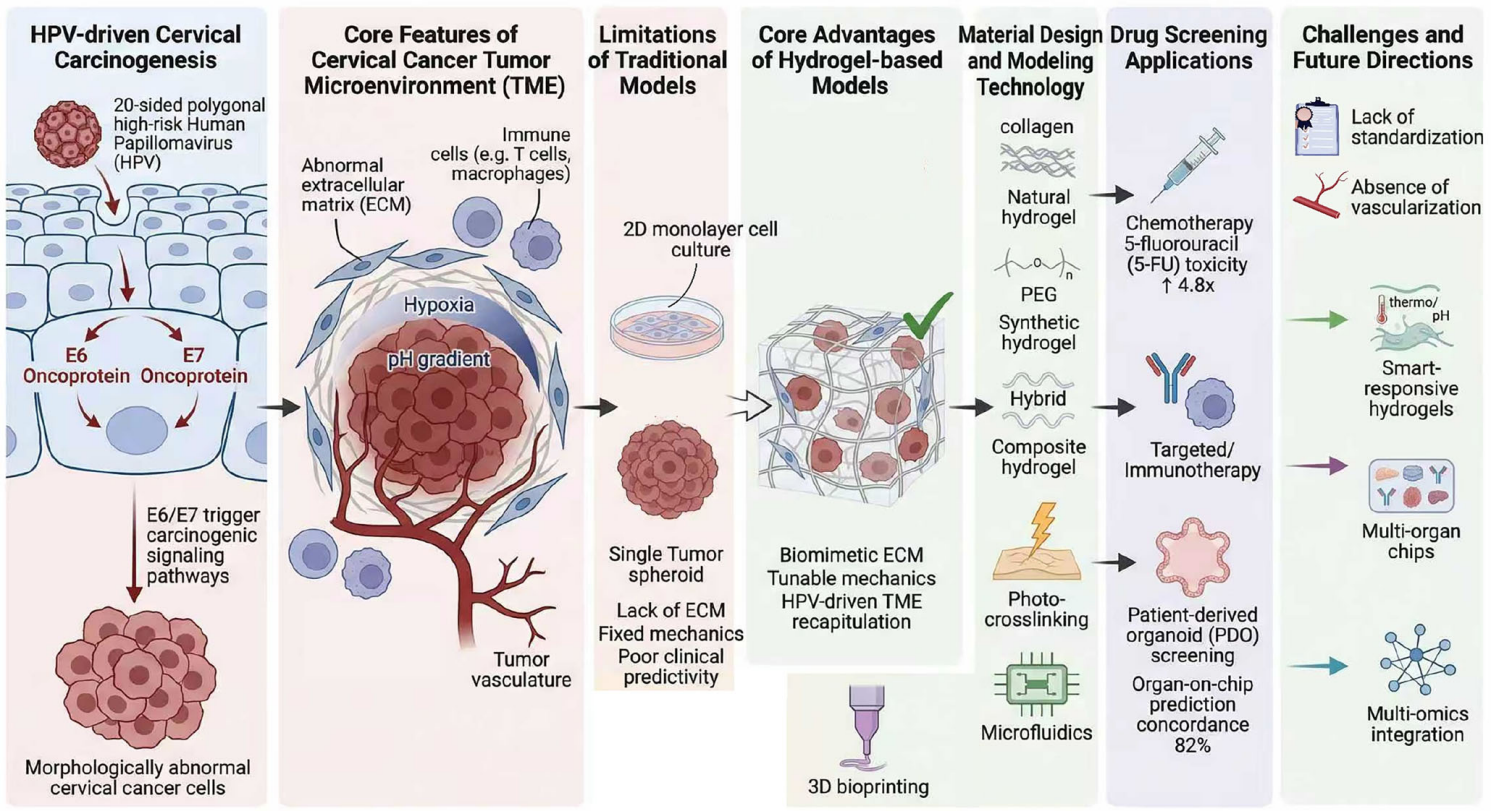
Overview of the full research landscape. This figure offers a panoramic overview of the entire research landscape, seamlessly linking the full research chain, from HPV‐driven cervical carcinogenesis, the core features of the cervical cancer tumor microenvironment (TME), limitations of traditional models, core advantages of hydrogel‐based models, material design and modeling techniques, and drug screening applications, to existing challenges and future directions.^2^, ^12^, ^13^, ^14^, ^15^, ^54^, ^65^ (1) HPV‐driven cervical carcinogenesis: illustrates the core mechanism by which high‐risk human papillomavirus (HPV) infects cervical epithelial basal cells, activates oncogenic signaling pathways via E6/E7 oncoproteins, and ultimately induces morphological abnormalities in cervical cells leading to cervical cancer. (2) Core features of cervical cancer TME: presents the core components of the cervical cancer tumor microenvironment (TME), including abnormal extracellular matrix (ECM), hypoxia and pH gradients, immune cells (T cells, macrophages), and tumor vascular networks, highlighting the spatial heterogeneity and functional characteristics of the cervical cancer TME. (3) Limitations of traditional models: compares the core deficiencies of 2D monolayer cell culture and simple tumor spheroid models, including lack of ECM scaffold, fixed mechanical properties, inability to fully recapitulate TME, and poor clinical predictivity, emphasizing that traditional models cannot meet the demands of precise cervical cancer research. (4) Core advantages of hydrogel‐based models: Clarifies the core value of hydrogel‐based 3D models, including biomimetic ECM structure, tunable mechanical properties, and full recapitulation of HPV‐driven TME features, positioning them as more physiologically relevant tools for cervical cancer research. (5) Material design and modeling techniques: Displays the classification of hydrogel materials (natural/synthetic/composite hydrogels) and core fabrication techniques (photocrosslinking, 3D bioprinting, microfluidics), providing technical support for model construction. (6) Drug screening applications: demonstrates the core applications of hydrogel models in cervical cancer drug screening, including key quantitative results such as a 4.8‐fold increase in selective cytotoxicity of 5‐fluorouracil (5‐FU), targeted/immunotherapy evaluation, patient‐derived organoid (PDO) screening, and 82% concordance of organ‐on‐chip toxicity prediction. (7) Challenges and future directions: highlights the core bottlenecks of the current field (lack of standardization, absence of vascularization) and prospects three cutting‐edge development directions: smart‐responsive hydrogels, multi‐organ chips, and multi‐omics integration. HPV, human papillomavirus; TME, tumor microenvironment; ECM, extracellular matrix; PEG, polyethylene glycol; 5‐FU, 5‐Fluorouracil; PDO, patient‐derived organoid.

Nevertheless, hydrogel‐based 3D cervical cancer models are still in the preliminary stage with critical limitations, including insufficient TME complexity, lack of standardization, and limited clinical translatability. Future advances must address vascularization, immune integration, and patient‐derived model construction to truly bridge preclinical testing and clinical outcomes (Figure 6).

## AUTHOR CONTRIBUTIONS

Ying Liu was responsible for writing the manuscript and investigation of the subject, such as conducting literature reviews to understand the current state of research. The author has read and approved the manuscript. Data authentication is not applicable.

## FUNDING INFORMATION

This study was provided financial support from the following projects: Jinzhou Medical University's 2025 Project for Educational Teaching Research and Reform (Grant No. YD2025013).

## CONFLICT OF INTEREST STATEMENT

The author declares no conflicts of interest.

## Data Availability

Data sharing not applicable to this article as no datasets were generated or analysed during the current study.
